# Effectiveness of The Umbrella Collaboration Versus Traditional Umbrella Reviews for Evidence Synthesis in Health Care: Protocol for a Validation Study

**DOI:** 10.2196/67248

**Published:** 2025-04-14

**Authors:** Beltran Carrillo, Marta Rubinos-Cuadrado, Jazmin Parellada-Martin, Alejandra Palacios-López, Beltran Carrillo-Rubinos, Fernando Canillas-Del Rey, Juan Jose Baztán-Cortes, Javier Gómez-Pavon

**Affiliations:** 1 The Umbrella Collaboration Madrid Spain; 2 Hospital Universitario Cruz Roja Madrid Spain; 3 Universidad Alfonso X el Sabio Villanueva de la Cañada, Madrid Spain

**Keywords:** tertiary evidence synthesis, The Umbrella Collaboration, umbrella reviews, health research methodology, AI-assisted synthesis, AI-assisted, evidence-based decision making, machine learning, ML, artificial intelligence, AI, algorithms, models, analytics, digital health, digital technology, digital interventions

## Abstract

**Background:**

The synthesis of evidence in health care is essential for informed decision-making and policy development. This study aims to validate The Umbrella Collaboration (TU), an innovative, semiautomatic tertiary evidence synthesis methodology, by comparing it with Traditional Umbrella Reviews (TUR), which are currently the gold standard.

**Objective:**

This study aimed to evaluate whether TU, an artificial intelligence—assisted, software-driven system for tertiary evidence synthesis, can achieve comparable effectiveness to TURs, while offering a more timely, efficient, and comprehensive approach. In addition, as a secondary objective, the study aims to assess the accessibility and comprehensibility of TU’s outputs to ensure its usability and practical applicability for health care professionals.

**Methods:**

This protocol outlines a comparative study divided into 2 main parts. The first part involves a quantitative comparison of results obtained using TU and TURs in geriatrics. We will evaluate the identification, size effect, direction, statistical significance, and certainty of outcomes, as well as the time and resources required for each methodology. Data for TURs will be sourced from Medline (via PubMed), while TU will use artificial intelligence—assisted informatics to replicate the research questions of the selected TURs. The second part of the study assesses the ease of use and comprehension of TU through an online survey directed at health professionals, using interactive features and detailed data access.

**Results:**

Expected results include the assessment of concordance in identifying outcomes, the size effect, direction and significance of these outcomes, and the certainty of evidence. In addition, we will measure the operational efficiency of each methodology by evaluating the time taken to complete projects. User perceptions of the ease of use and comprehension of TU will be gathered through detailed surveys. The implementation of new methodologies in evidence synthesis requires validation. This study will determine whether TU can match the accuracy and comprehensiveness of TURs while offering benefits in terms of efficiency and user accessibility. The comparative study is designed to address the inherent challenges in validating a new methodology against established standards.

**Conclusions:**

If TU proves as effective as TURs but more time-efficient, accessible, and easily updatable, it could significantly enhance the process of evidence synthesis, facilitating informed decision-making and improving health care. This study represents a step toward integrating innovative technologies into routine evidence synthesis practice, potentially transforming health research.

**International Registered Report Identifier (IRRID):**

PRR1-10.2196/67248

## Introduction

### Background

The synthesis of evidence in health care is a knowledge-acquisition process designed to transform extensive volumes of data into manageable information to support decision-making based on the best possible evidence. It aims to integrate information from multiple sources on complex topics in a comprehensive, precise, transparent, and easily understandable manner. These principles ensure that synthesized evidence is accessible and useful to all stakeholders, including health care professionals, policy makers, and patients [1].

Evidence synthesis plays a pivotal role in knowledge translation (KT) [2], serving as a bridge between research and health care. Consequently, evidence synthesis is essential for the development of health policies and informed decision-making [3].

The role of all stakeholders in health decision-making underscores the importance of their ability to access, understand, and evaluate health information adequately [4]. A persistent challenge in KT is the low level of statistical and health literacy among the general population and health professionals, which significantly complicates effective health management [5,6]. Therefore, it is crucial to democratize access to high-level health information and to promote active participation of all stakeholders in decisions affecting health care [7].

The evolution of evidence synthesis methodologies has led to the development of tertiary synthesis, designed to condense knowledge from multiple systematic reviews with or without meta-analyses (SR/MA). This synthesis, often referred to as Umbrella Reviews now referred to as Traditional Umbrella Reviews (TUR) to distinguish them from the experimental methodology under study, The Umbrella Collaboration (TU), builds upon the concept of primary studies (individual studies with participant samples) and secondary studies (systematic reviews and analysis of those primary studies). Tertiary synthesis represents a third level, named with other terms such as overviews, meta-epidemiological studies, meta-analyses, meta-synthesis, and meta-reviews also describing this approach [8,9]. Tertiary synthesis has gained prominence in contexts where broad research questions are posed, rapid results are needed, and resources for extensive systematic reviews are limited. TURs follow a structured methodological process that involves several clearly defined stages [9-14]. Although organizations like Cochrane Collaboration [14] and the Joanna Briggs Institute (JBI) [11] have developed and continually updated detailed methodologies for these reviews, there remains considerable divergence in how TUR authors implement these steps in practice. This methodological freedom leads to significant variations among different TURs in terms of rigor and approach.

The urgency for high-quality, timely information during crises like the SARS-CoV-2 pandemic has highlighted the critical need for faster evidence synthesis methods, even if it means accepting certain limitations in comprehensiveness, detail, and precision [15]. This demand has driven the development of innovative approaches such as TU, which leverages artificial intelligence (AI)-assisted software to facilitate tertiary evidence synthesis under human oversight. While AI tools such as PICO (Population, Intervention, Comparison, and Outcome) Portal, DistillerSR, Covidence (Veritas Health Innovation), and Rayyan (Rayyan Systems Inc) have already improved secondary evidence synthesis by streamlining data management and analysis [16], technologies like large language models (LLMs), including ChatGPT, are beginning to demonstrate potential for conducting systematic reviews autonomously, though human supervision remains essential to mitigate risks such as errors and hallucinations [17]. Despite these developments in secondary synthesis, the application of AI and software engineering in tertiary synthesis is still in its early stages, with no dedicated software currently available. TU is at the forefront of this field, pioneering the integration of AI and software engineering with human oversight to ensure accuracy and minimize technology-induced errors. As AI continues to evolve, it is likely that fully automated processes for both secondary and tertiary synthesis will emerge, potentially revolutionizing clinical research and practice. However, building confidence in these technologies will require ongoing development and rigorous validation.

### The Umbrella Collaboration (Patent Pending)

TU is primarily a software-driven system engineered to streamline tertiary evidence synthesis, relying on programmed algorithms to automate the majority of its functions. The core of the system is built on a software infrastructure that processes and synthesizes data from SR/MA abstracts stored in MEDLINE. While AI plays a crucial role, particularly through the use of LLMs and machine learning (ML), it is used selectively within the broader software framework to enhance specific tasks.

LLMs are used in generating related search terms, expanding upon human-generated queries to enhance the comprehensiveness of literature searches. Any LLM can be adapted to TU software, up to date we have used ChatGPT 4 [18]. This function is crucial in broadening the scope of the searches while ensuring that the results remain relevant and precise. The AI component is designed to support, not replace, human oversight, ensuring that the final selection of literature is both accurate and comprehensive [19]. To mitigate the risk of AI-generated hallucinations due to insufficient data, TU primarily operates as a stable, auditable software system. The use of AI is limited to the search term expansion phase, where it suggests synonymous terms for the keywords provided by the human reviewer.

All AI-generated search terms are subject to human validation before being incorporated into the search strategy. The human reviewer evaluates the relevance and appropriateness of each AI-suggested term, ensuring that only those that align with the research objectives are retained. This manual oversight serves as a critical safeguard against inaccuracies or misleading AI-generated suggestions, maintaining the methodological integrity of the evidence synthesis process.

As the TU database grows, ML will be integrated to further refine and optimize the software’s performance. Training the system on an expanding dataset is expected to enhance its ability to select, categorize, and analyze relevant research, thereby improving both efficiency and accuracy over time. This approach allows TU to evolve, continuously improving its utility in evidence synthesis through the iterative learning process [20].

Overall, TU represents a hybrid model where traditional software engineering and targeted AI applications work in tandem. This balance ensures that while the software performs most functions automatically, AI enhances specific tasks, such as search term generation and future predictive analysis, under strict human supervision. This strategic integration of AI elements within a primarily software-driven system ensures the reliability and precision of the evidence synthesis process.

The outcomes generated by TU will be presented through an interactive web application designed to enhance accessibility and comprehension for a broad range of stakeholders, including those without advanced statistical expertise. The use of graphical formats and clear language aims to facilitate the interpretation of findings by diverse audiences.

In addition, the platform supports continuous updates, automatically integrating new SR/MA data every 24 hours, thereby ensuring the most current and reliable evidence synthesis. This approach aligns with the concept of Living Systematic Reviews (LSRs) [21], which advocate for frequent updates to maintain relevance in rapidly evolving fields. While Cochrane Collaboration recommends updating LSRs monthly [22], TU is designed to surpass this standard by ensuring updates are incorporated daily, providing near real-time evidence synthesis.

The daily updates provided by TU are expected to enhance its efficiency, allowing for a more dynamic and continuously updated evidence synthesis process. Pilot tests have demonstrated TU’s capability to complete tertiary evidence synthesis projects within hours, a significant reduction in time compared to traditional methods (unpublished data). If validated by the upcoming research, this advancement could demonstrate TU’s potential to streamline the synthesis process, delivering rapid yet reliable results while upholding the highest standards of accuracy. Should these findings be confirmed, TU may emerge as an invaluable tool for accelerating the pace of evidence-based research.

TU is designed to maximize computational efficiency while maintaining methodological rigor. Unlike TURs, which require extensive manual data extraction and synthesis over months, TU automates critical steps in the tertiary synthesis process, significantly reducing execution time.

In terms of computational complexity, TU does not perform direct statistical meta-analyses but instead extracts and synthesizes pre-existing systematic reviews and meta-analyses. This approach ensures that the computational load remains minimal compared to methodologies that require full-scale meta-analyses or real-time data processing. The most computationally intensive process within TU is the search term expansion using AI, which is constrained to generating synonymous terms based on human-input keywords. This AI function operates on a lightweight model that does not require high-performance computing resources.

A key distinction of TU is that it assists in the entire tertiary review process, rather than being limited to isolated stages. Compared to existing approaches such as DistillerSR, Covidence, and Rayyan, which focus on specific tasks like study screening or data extraction, TU integrates a complete methodology for tertiary evidence synthesis. This includes search term expansion, literature retrieval, synthesis automation, and structured result visualization. While other tools provide assistance in certain steps, TU ensures a fully automated, structured, and reproducible workflow for umbrella reviews.

To our knowledge, there are currently no automated systems specifically designed for tertiary evidence synthesis that comprehensively address and assist in the entire synthesis process. While several tools exist for systematic review automation—such as DistillerSR, Covidence, and Rayyan—these primarily focus on secondary evidence synthesis (ie, systematic reviews) and are not designed to facilitate tertiary synthesis methodologies like umbrella reviews.

In addition, no previous tool has undergone a formal assessment or validation for automated tertiary synthesis, as TU is the first system explicitly developed for this purpose. Unlike existing software, TU does not merely automate isolated steps (such as literature screening or data extraction) but provides a structured, end-to-end approach for tertiary evidence synthesis. This distinction highlights the novelty of TU and underscores the need for this study to formally assess its performance compared to TURs.

A schematic diagram is provided to clearly illustrate TU’s workflow, showing the process from data acquisition to the generation of synthesized results. Abstracts of SR/MA, retrieved from the MEDLINE database via PubMed, form the foundation of the analysis. The decision to use MEDLINE via PubMed as the sole database for literature retrieval in TU is based on its strong coverage of systematic reviews and meta-analyses, as well as the feasibility of leveraging LLMs for search term expansion. Recognizing the potential limitation of relying on a single database, we conducted preliminary assessments to estimate the impact of this decision. Specifically, we evaluated the ability of TU to retrieve references from TURs and analyzed the proportion of systematic review and meta-analysis references found in MEDLINE. Our results showed that TU was able to retrieve 81.1% (414/511) of TUR references using its AI-assisted search methodology in Medline alone. In addition, an independent assessment of 511 references from 22 TURs found that only 11 references were not indexed in MEDLINE. While some loss of relevant studies is inevitable, these findings suggest that the methodological approach used in TU remains sufficiently comprehensive for tertiary evidence synthesis, balancing feasibility and completeness (unpublished data). The data obtained are processed through a range of techniques, including natural language processing (NLP), sentiment analysis (SA), web scraping (WS), and ML. The expected results comprise synthesized evidence on intervention effectiveness and risk exposures, presented in a graphical and visual format. These results are conveyed in plain language, making them easily understandable by all stakeholders, regardless of their statistical literacy.

**Figure 1:**
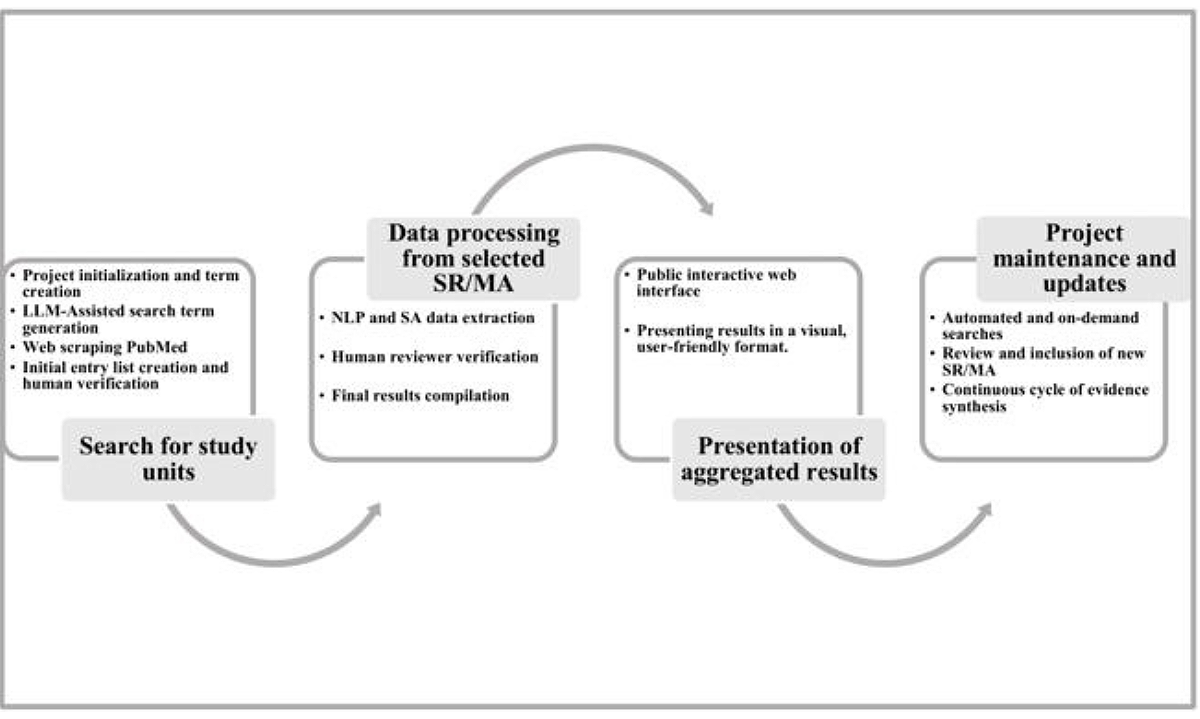
The Umbrella Collaboration workflow. NLP: natural language processing; LLM: large language models; SA: sentiment analysis; SR/MA: systematic reviews with or without meta-analyses.

The project is continuously updated through automated and on-demand searches, with data from new studies seamlessly integrated into the existing body of evidence. Each inclusion restarts the synthesis process, creating a dynamic, cyclical workflow that ensures the results of the project remain up-to-date (Figure 1).

The implementation of new methodologies in the scientific field requires a comparative validation process with established methods to ensure their reliability and effectiveness. TU, being an innovative methodology still in its theoretical-conceptual stage, must be evaluated against established methodologies. Therefore, the aim of this study is to validate TU by comparing its performance and outcomes with the gold standard, TURs, to establish its credibility and potential superiority.

### Objectives

The primary objective of this study is to assess whether a software-driven AI-assisted system of evidence synthesis, TU, can match the effectiveness of traditional methods of tertiary synthesis, providing a potentially more timely, efficient, and comprehensive approach while remaining open to findings that could demonstrate superior performance. To support the primary objective of evaluating the effectiveness of TU compared to traditional methodologies, this study also aims to assess the accessibility and comprehensibility of TU’s outputs as a secondary objective.

## Methods

### Study Design

#### Part 1: Quantitative Comparison of Methodologies

##### Overview

**Figure 2:**
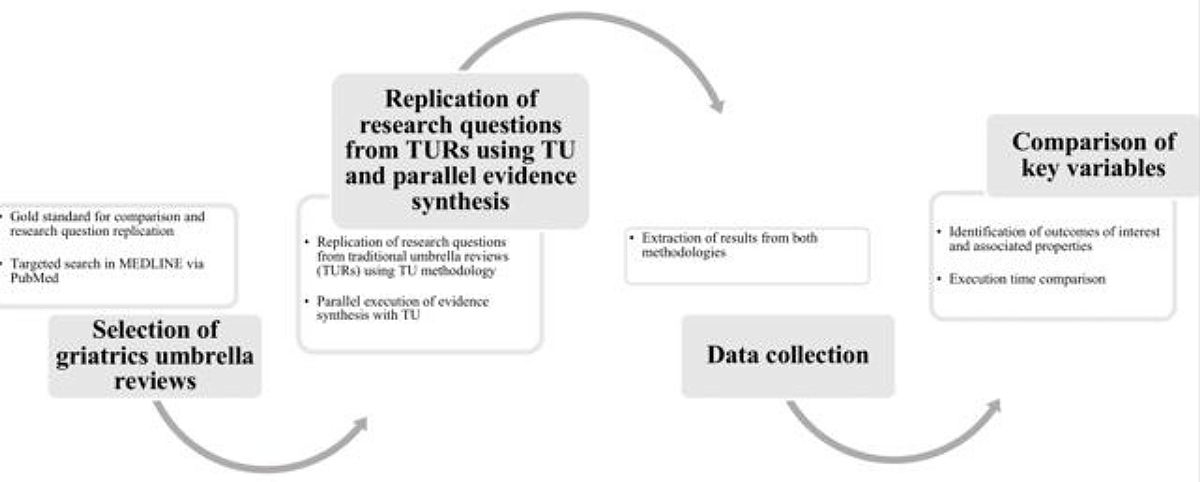
Study design overview. TU: The Umbrella Collaboration; TUR: Traditional Umbrella Reviews.

Figure 2 provides an overview of the study design. The study follows a structured comparative approach in which selected TURs in geriatrics serve as the gold standard for validation. Using the same research questions, projects are conducted in parallel with TU to assess its performance. Data from both methodologies are systematically collected and compared, focusing on key variables such as the identification of outcomes of interest, effect size, effect direction, statistical significance, certainty of evidence, and execution time. The figure illustrates the step-by-step workflow of the study, from the selection of umbrella reviews in geriatrics as reference models to the comparison of results obtained using TU and TURs.

The first part of this study focuses on a quantitative comparison between the 2 tertiary synthesis methodologies. To facilitate this comparison, a targeted search in PubMed will identify relevant TURs in geriatrics, focusing on representative reviews rather than an exhaustive search. Our approach will involve a focused search in PubMed, using specific terms relevant to geriatrics, to find suitable TURs that serve as a benchmark for this comparative analysis. This targeted search is sufficient for our methodological comparison and does not require the comprehensive search strategy typical of systematic reviews, as our goal is not to cover the entire scope of available literature but to enable a parallel evaluation of synthesis methodologies. Therefore, while the search strategy may appear basic, it is intentionally designed to fulfill the specific needs of our project without aiming for exhaustive literature retrieval, which is beyond the scope of this project.

##### Study Variables

In the quantitative comparison, several critical variables will be analyzed. Key among these is the identification and evaluation of outcomes of interest (outcomes). This includes assessing the degree of concordance between the methodologies in identifying outcomes and using a concordance matrix to document and compare the outcomes identified by TU and TURs. In addition, we will analyze the total number of outcomes identified by each methodology, providing a descriptive and statistical comparison. It is essential to define the concept of an “outcome of interest” within the context of tertiary evidence synthesis. An outcome of interest refers to specific aspects identified and evaluated by systematic reviews with meta-analyses that examine the same research question. These outcomes are critical for understanding the overall impact of various interventions on health conditions or the effects of exposure to risks.

A crucial aspect of this analysis involves the comparison of effect sizes for the identified outcomes. TU uses an automated approach for standardizing effect sizes using a custom-designed metric (R_TU_). This metric transforms all commonly used effect size statistics in evidence synthesis (eg, standardized mean difference [SMD], mean difference [MD], relative risk [RR], odds ratio [OR], hazard ratio [HR], and others) into a single weighted composite measure. The R_TU_ metric enables the aggregation of heterogeneous effect size measures across different systematic reviews supporting a given outcome of interest in tertiary evidence synthesis.

Since this process is fully automated, accuracy is assessed through comparisons with TURs. By replicating research questions from TURs in TU, we compare whether the R_TU_-derived effect sizes align with those obtained using traditional methodologies. This validation step ensures that the automated transformation process does not introduce systematic distortions and maintains consistency with established effect size estimation methods. Given the diverse metrics used in TURs, such as SMD, MD, RR, OR, and HR, and the unique metric used by TU, we will standardize all effect sizes, used in TURs, to Cohen *d*. This standardization facilitates a direct comparison, ensuring consistency in the interpretation of results.

We will also examine the direction of the effects for each outcome, categorizing them as favorable, unfavorable, or unknown for interventions, and as increasing, decreasing, or unknown for exposures to risks. The statistical significance of the outcomes will be compared by analyzing *P* values and CI, assessing whether the results are statistically significant across both methodologies.

Furthermore, the certainty of the evidence (LoE) associated with each outcome will be evaluated. To assess the LoE within TU, we use sentiment analysis as a NLP technique. This approach allows for the automated classification of certainty indicators extracted from systematic review abstracts. Regarding the concern about the training data for sentiment analysis, at this stage, TU uses a sentiment analysis model initially trained on X (formerly known as Twitter) data. While we acknowledge that this is not highly optimized for medical texts, it provides a cost-effective starting point for sentiment classification without incurring additional expenses. Cloud-based solutions, such as Azure cognitive services, offer more specialized sentiment analysis models for health care, but these are paid services that exceed our current budget.

We fully recognize the limitations of using general sentiment analysis for LoE, as traditional GRADE (Grading of Recommendations Assessment, Development, and Evaluation)-based approaches consider multiple factors such as study limitations, inconsistency, indirectness, publication bias, and confounders. However, our primary aim is to determine whether a simplified approach, using abstract-level sentiment classification, can yield reasonable agreement with traditional methodologies. TU does not claim to replace the rigor of full-text GRADE assessments but instead seeks to evaluate whether an alternative automated method can provide valuable insights with lower resource demands. We are actively working toward developing our own custom sentiment analysis algorithm tailored to medical literature, trained on validated medical datasets. This will enhance precision and improve TU’s ability to evaluate LoE more effectively in the future.

For TURs, this will be done using the GRADE system [23], which categorizes evidence into very low, low, moderate, and high levels. TU applies an SA-based scoring system on a scale from –1 to +1. Both scales will be normalized to a similar quantitative range (0-1) to facilitate comparison. To align the 2 systems, the SA scores are normalized using the following transformation formula: X=(SA score+1)/2. Where X represents the normalized certainty score, ensuring that SA values originally in the range of –1 to 1 are mapped to a 0 to 1 scale. The GRADE ordinal levels are mapped onto the 0-1 scale as follows: very low=0.00-0.25, low=0.26-0.50, moderate=0.51-0.75 and high=0.76-1.00. This process allows TU and TUR certainty ratings to be compared in a standardized manner, enabling statistical concordance analyses between the 2 methodologies.

Finally, the execution time of each methodology will be assessed, with TU providing exact time measurements and TURs relying on an estimated timeframe of 6 to 12 months based on existing literature.

##### Data Collection and Research Question Replication

Data collection will begin with a targeted search in PubMed to identify TURs in geriatrics, using the search terms “umbrella” AND “geriatric.” The identified TURs will serve as benchmarks for our comparative analysis. The research questions from these selected umbrella reviews will be directly replicated in TU without modification, ensuring a precise comparison of outcomes generated by each methodology. TU will be configured to replicate these questions, using automated searches and synthesis through NLP, WS, SA, and ML, with human reviewers verifying and extracting data as necessary. This approach allows us to assess the comparative effectiveness and efficiency of TU relative to traditional methods, particularly in identifying and analyzing outcomes critical to evaluating health interventions and exposure risks. Data from both TURs and TU will be systematically collected and recorded in a database to facilitate precise comparisons of outcomes, effect sizes, and other critical variables, ensuring a thorough evaluation of both methodologies.

The decision to rely solely on abstracts for data extraction in TU was driven by practical and methodological considerations. First, this approach was chosen due to budgetary constraints, as full-text access to all systematic reviews and meta-analyses would require extensive licensing fees or institutional subscriptions, which are beyond the scope of this project. Second, abstracts help mitigate language bias, since all systematic review abstracts indexed in MEDLINE are available in English, regardless of the original language of publication. This ensures a broader and more internationally representative evidence base.

While it is acknowledged that abstracts often contain limited methodological details and may lack comprehensive information on certainty of evidence, outcome effect sizes, or risks of bias, the core objective of TU methodology is to assess whether robust conclusions can still be drawn based on abstracts alone. Recognizing the inherent limitations of abstracts, TU incorporates structured extraction criteria to capture the most relevant information while acknowledging the potential risks of missing key methodological details. The validation process of TU explicitly includes a comparison with full-text TURs to evaluate whether synthesis based on abstracts alone yields comparable conclusions.

#### Part 2: Evaluation of Ease of Use and Comprehension

##### Overview

The second part of the study focuses on evaluating and comparing the ease of use and comprehension of the results generated by TU with those from TURs. This evaluation will be conducted through an anonymous and voluntary online survey directed at health professionals, designed to assess their experience with both methodologies.

The survey, developed using Google Surveys for ease of access and data analysis, comprises 16 items. The initial 6 items gather demographic information about the survey respondents, while the subsequent 10 questions directly compare the usefulness and clarity of the results produced by TU and TURs, using a Likert scale ranging from 1 to 5. This scale will measure respondent’s perceptions of the clarity, comprehensibility, and ease of use of the results provided by both methodologies (Table 1).

To ensure a thorough evaluation, informational sessions will be held in geriatric departments of university hospitals in Madrid (Spain). During these sessions, the concept of tertiary evidence synthesis and the functionality of TU will be introduced. Health professionals, including those from geriatrics and other rotating specialties, will be given access to the TU platform and guided through its use by an expert. This hands-on experience will be complemented by providing the participants with TUR result tables to facilitate a direct comparison.

Participants will be able to access the survey via a QR code, provided during the sessions, allowing them to complete it either immediately or at their convenience. The survey’s responses will be analyzed descriptively, focusing on the overall user experience with TU and its potential advantages in terms of ease of interpretation and presentation compared to traditional methods.

**Table 1:** Survey questions on the utility of The Umbrella Collaboration.

	Question	Answer Likert scale
1 - 6	Respondent affiliation data	
7	Do you consider that the interactive interface of the “TU”^a^ methodology facilitates the understanding of results compared to the static tables of TURs^b^?	1: Strongly disagree 2: 3: 4: 5: Strongly agree
8	⁠Do you believe that the visualization of results using bubble plots in “TU” helps to quickly identify the most relevant outcomes?	1: Strongly disagree 2: 3: 4: 5: Strongly agree
9	Does the graphical representation of the outcomes (illustrated by figures, colors, and sizes) in “TU” enhance your ability to assess the clinical relevance of the results?	1: Strongly disagree 2: 3: 4: 5: Strongly agree
10	Do you find that access to detailed data by clicking on the figures of the outcomes in “TU” interface enhances your evidence analysis experience?	1: Strongly disagree 2: 3: 4: 5: Strongly agree
11	Do you think that “TU” methodology allows for a quicker interpretation of data compared to TUR?	1: Strongly disagree 2: 3: 4: 5: Strongly agree
12	Is the information provided by “TU” useful in your field of work (clinical, research, or educational) for evidence-based decision-making?	1: Strongly disagree 2: 3: 4: 5: Strongly agree
13	Does the ease of use of “TU” interface facilitate greater data exploration compared to traditional methods?	1: Strongly disagree 2: 3: 4: 5: Strongly agree
14	Do you consider the historical evolution of evidence provided by “TU” methodology to be useful?	1: Strongly disagree 2: 3: 4: 5: Strongly agree
15	Does “TU” methodology require less statistical knowledge to interpret the results compared to TUR?	1: Strongly disagree 2: 3: 4: 5: Strongly agree
16	Overall, are you satisfied with “TU” methodology as a tool for tertiary evidence synthesis?	1: Strongly disagree 2: 3: 4: 5: Strongly agree

^a^TU: The Umbrella.

^b^TURs: Traditional Umbrella Reviews.

##### Statistical Analysis: Data Analysis and Statistical Methods

The quantitative analysis will compare the results obtained by both methodologies. Contingency tables will be constructed to contrast the identification of outcomes, the size effect, the direction of the effect, and the statistical significance, ensuring statistical congruence between the methods. For the evaluation of certainty levels, TU scores will be normalized to a scale comparable to the GRADE levels, which will also be transformed into a numerical scale between 0 and 1.

The chi-square test will be used to determine significant differences between the methodologies. In addition, Pearson and Spearman correlation analyses will be conducted to quantify the relationship between TU certainty levels and GRADE certainty levels of the TURs. Pearson correlation analysis is useful for quantifying the strength and direction of a linear relationship between 2 continuous variables. Moreover, the analysis will be complemented with Spearman correlation analysis due to potential violations of normality assumptions in the data.

Statistical analysis will be performed using IBM SPSS Statistics (version 26), with an alpha level of .05 to determine statistical significance.

### Ethical Considerations

This study does not involve human participants, personal data, or animals. All data will be sourced from published materials, and the analyses will be conducted in accordance with established ethical standards for secondary data analysis. In addition, the survey component will be conducted anonymously and on a voluntary basis. Given these considerations, we do not deem it necessary to seek approval from an ethics committee, as the study adheres to standard ethical practices for research of this nature [24].

## Results

We will focus on evaluating the effectiveness of the experimental methodology in accurately identifying and analyzing relevant outcomes. Among the results, we will include detailed assessments of effect sizes, the direction of effects, statistical significance, and the certainty of evidence for each outcome. We will compare these findings with those derived from TUR to determine if the experimental approach yields results that are at least equivalent in quality and comprehensiveness.

Finally, the efficiency of the experimental methodology will be evaluated by recording the time taken to complete the synthesis process. While the time required for TUR is estimated to range between 6 and 12 months based on existing literature, we will document the actual time taken by the experimental approach. This will provide a practical measure of the potential time savings offered by the software-driven AI-assisted method, highlighting its feasibility and effectiveness in a real-world context.

To evaluate the ease of use and comprehension of TU within its environment, a detailed survey will be specifically designed. Survey respondents will have access to the TU platform, where they can interact with various interactive screens displaying the results of the synthesis process across different projects completed to date.

## Discussion

### Principal Findings

The anticipated findings of this study are expected to demonstrate that TU, a semiautomated tertiary evidence synthesis tool, can produce results that are methodologically comparable to TURs while significantly improving efficiency. By leveraging AI-assisted methodologies, TU may streamline the synthesis process, reducing the time and effort required for evidence aggregation. In addition, the study aims to assess whether tertiary evidence synthesis can be effectively conducted using only systematic review abstracts, balancing feasibility with methodological rigor. If successful, these findings could support the broader adoption of AI-driven approaches in tertiary synthesis, potentially transforming the landscape of evidence-based decision-making in health care.

The primary objective of this study is to evaluate whether TU, a software-driven system designed to facilitate tertiary evidence synthesis with AI-assisted methodologies, can match the effectiveness of TURs. TU integrates with its software development advanced technologies such as NLP, SA, WS, and ML to enhance the efficiency of evidence synthesis.

The semiautomated processes implemented by TU could signify a significant advancement in making evidence synthesis more accessible and timely, with the potential for continuous updates as new data becomes available. The study’s findings could pave the way for broader adoption of AI-driven methodologies in evidence synthesis, potentially reducing the time and resources needed for comprehensive reviews. TU’s integration of software engineering projects and AI with traditional methods could streamline the review process, enabling faster aggregation and interpretation of data across various research domains.

Several software tools assist in systematic and umbrella review processes, such as DistillerSR, Covidence, and Rayyan. These platforms improve efficiency in evidence synthesis by automating tasks such as study screening, data extraction, and literature management. However, they do not offer a fully structured and automated methodology for tertiary evidence synthesis, which remains a predominantly manual process. In contrast, TU is designed to support the entire tertiary synthesis workflow, rather than focusing on isolated stages. TU integrates (1) AI-assisted search term expansion, which enhances literature retrieval by suggesting synonymous terms for human validation; (2) automated data extraction from systematic review abstracts, allowing for a structured and efficient synthesis process; (3) a dedicated framework for tertiary synthesis, unlike other tools that primarily assist in secondary synthesis (systematic reviews); and (4) interactive and visual result presentation, providing stakeholders with an accessible interpretation of findings, rather than traditional tabular outputs.

### Ongoing Project Status

Once created, projects in TU remain active indefinitely, preventing obsolescence. This continuous updating of evidence synthesis is a key feature that differentiates TU from static review methodologies. The system conducts literature searches every 24 hours or on demand by the human reviewer responsible for the project. This allows for real-time incorporation of newly published studies, ensuring that the synthesis remains current and reflective of the latest scientific evidence. While TUR requires manual updates, often years apart, TU transforms the synthesis process into a living evidence system, ensuring that the latest research is incorporated seamlessly. This eliminates the need for reinitiating entire projects and allows researchers to work with continuously updated data.

One similarity between TU and existing platforms is the incorporation of automation to enhance efficiency. However, TU distinguishes itself by providing a fully integrated tertiary synthesis methodology with a self-updating mechanism, ensuring both methodological rigor and computational efficiency in umbrella reviews.

By offering a platform that supports real-time updates and provides accessible synthesis outputs, TU has the potential to enhance the utility of tertiary synthesis for a wide range of stakeholders, including those with limited statistical expertise. However, a key limitation of TU is its reliance on a single database (MEDLINE via PubMed), which might not capture all relevant studies. This study will examine whether this limitation can be mitigated by the system’s other capabilities. Ultimately, the timely and comprehensible evidence synthesis provided by TU could facilitate more informed decision-making in clinical settings, particularly in rapidly evolving areas of medical research.

Future research will be essential to further validate and refine TU. Planned next steps include expanding the sample size of included TURs to increase representativeness, conducting blinded validation studies to minimize potential biases, and testing the tool across different reviewer profiles to assess usability beyond expert users. In addition, TU’s methodology should be applied to other medical and nonmedical disciplines to evaluate its versatility. A critical aspect of future validation will also involve independent expert review, ensuring that TU’s findings align with established methodological standards. Finally, economic feasibility studies comparing TU with traditional synthesis approaches will be necessary to assess its cost-effectiveness and scalability in real-world implementation.

### Limitations

The decision to use only one database, MEDLINE via PubMed, in TU is both a recognized limitation and a deliberate choice shaped by resource constraints and technical considerations. While systematic reviews typically require searching multiple databases to capture all relevant literature [25], our approach focuses on testing whether TU can achieve outcomes comparable to TURs despite these limitations. While the pilot study provided preliminary evidence of PubMed’s strong coverage, further validation through TU is necessary to confirm its applicability across different domains.

Furthermore, while searching multiple databases is often recommended to avoid language and indexing biases, especially those related to non-English literature [26,27]. TU mitigates some of these biases by focusing on abstracts in English, as all PubMed abstracts are provided in this language regardless of the original publication’s language. However, the absence of Chinese databases in our approach is a notable limitation, given that only a small proportion of Chinese journals are indexed in MEDLINE.

The decision to rely exclusively on abstracts rather than full texts in TU is a deliberate methodological choice aligned with the core objective of this study: to evaluate whether tertiary evidence synthesis can be conducted efficiently, with fewer resources, and without requiring extensive methodological expertise from the reviewer, while still producing results comparable to TURs.

We acknowledge that abstracts may lack key methodological details, including certainty of evidence assessments, effect size calculations, and risk of bias evaluations. However, the fundamental hypothesis of TU is that an automated, structured approach to abstract-based synthesis may still yield clinically useful conclusions, particularly when applied under standardized and reproducible conditions. Moreover, relying on abstracts offers two key advantages: (1) minimization of language bias: all systematic review abstracts in MEDLINE are available in English, regardless of the original publication language, which ensures that non-English studies are not automatically excluded due to language barriers, a common issue in traditional systematic reviews; and (2) feasibility and accessibility: full-text access to all systematic reviews requires extensive licensing fees and institutional subscriptions, which may not always be feasible. Abstracts provide a universally accessible data source, allowing for broader implementation of evidence synthesis methodologies.

While TU does not claim to replace the depth of full-text review, this study aims to evaluate whether a structured synthesis based solely on abstracts can yield results that are sufficiently coherent and robust to serve as a complementary or alternative approach. If validated, this methodology could provide an efficient solution for synthesizing evidence in settings where full-text access is restricted or when rapid evidence synthesis is needed.

While we acknowledge the potential benefits of testing TU across multiple medical domains, this study is a pilot study designed to assess the feasibility of our methodology in a single, well-defined field, geriatrics. At this stage, limiting the scope to geriatrics is a strategic choice, ensuring that the study maintains clarity, feasibility, and methodological rigor in its initial validation phase. This focused approach allows for a controlled evaluation of TU against TURs, ensuring that the initial validation is conducted under clearly defined conditions. If the results demonstrate that TU can produce findings comparable to TURs, future studies will expand its application to other medical fields, such as cardiovascular medicine and psychiatry, as well as nonmedical domains, including education, sociology, and other disciplines with abundant systematic reviews. This stepwise approach ensures a methodologically sound progression, allowing TU to be tested and refined incrementally before broader implementation.

In this initial phase of the study, we have prioritized clinical health professionals as the primary participants for the ease of use and comprehension surveys. This decision is based on the primary objective of TU, which is to facilitate evidence synthesis for clinicians and health care professionals who may not have extensive expertise in systematic review methodologies. Given that TU is designed to enhance the accessibility and usability of tertiary evidence synthesis in clinical practice, it is essential to first evaluate its clinical utility and interpretability among end-users.

We fully acknowledge the importance of a rigorous methodological review by epidemiologists and research methodologists, as well as the need to assess the validity of results produced by TU. However, this will be addressed in a subsequent phase of research, where surveys will be extended to other key stakeholders, including epidemiologists and research methodologists, to evaluate the methodological robustness of TU. Health care policy makers and hospital administrators, assess TU’s potential role in decision-making. Patients and caregivers, to explore how synthesized evidence can be communicated effectively to the general population. This stepwise approach ensures that TU is first assessed from a clinical perspective before expanding to other critical stakeholders in future validation studies.

Another limitation of this study is the potential for response bias in the ease-of-use and comprehension survey. Given that most participants will complete the survey immediately after structured demonstrations of TU, their responses could be influenced by the context of the presentation. This could lead to a more favorable assessment of TU than what might be observed in an independent evaluation setting. This halo effect can impact the perceived usability and effectiveness of TU. Future studies should aim to validate these findings through independent assessments in settings where TU is used without direct guidance from the research team.

### Conclusion

This study aims to validate TU as a tool for tertiary evidence synthesis in health. If this methodology proves to be as effective as TURs, but more efficient in terms of project execution time and more accessible in terms of ease of use and comprehension, it could significantly enhance the way evidence synthesis is conducted, facilitating informed decision-making, and improving health outcomes. The results of this study may represent a step toward the integration of innovative technologies into the routine practice of evidence synthesis, with the potential to transform the field of health research.

## Abbreviations

AI: artificial intelligence
GRADE: Grading of Recommendations Assessment, Development and Evaluation
HR: hazard ratio
JBI: Joanna Briggs Institute
KT: knowledge translation
LLM: large language model
LoE: certainty of the evidence
LSR: Living Systematic Review
MD: mean difference
ML: machine learning
NLP: natural language processing
OR: odds ratio
PICO: Population, Intervention, Comparison, and Outcome
RR: relative risk
RTU: custom-designed metric
SA: sentiment analysis
SMD: standardized mean difference
SR/MA: systematic reviews with or without meta-analyses
TU: The Umbrella Collaboration
TUR: Traditional Umbrella Reviews
WS: web scraping
